# From Hemorrhage to Pyometra: A Complex Postpartum Cascade Following B-Lynch Suturing and Bilateral Internal Iliac Artery Ligation

**DOI:** 10.7759/cureus.116621

**Published:** 2026-09-21

**Authors:** Sangam Jha, Purnima Sagar, Pammy Pravina, Garima Prajapati, Vikas C Jha

**Affiliations:** 1 Obstetrics and Gynaecology, All India Institute of Medical Sciences, Patna, Patna, IND; 2 Neurosurgery, All India Institute of Medical Sciences, Patna, Patna, IND

**Keywords:** b-lynch, compression suture, internal iliac artery ligation, postpartum hemorrhage, uterine necrosis

## Abstract

Postpartum hemorrhage (PPH) due to uterine atony remains a major cause of maternal morbidity and mortality worldwide. When medical therapy fails, conservative surgical measures, such as uterine compression sutures and vascular ligation, are used to control bleeding while preserving fertility. Although generally effective, these procedures may rarely lead to severe complications, including uterine ischemia, infection, and necrosis. We report a case of a primiparous woman who developed primary PPH following normal vaginal delivery and required uterine compression sutures for hemostasis. Subsequently, she developed complete uterine necrosis necessitating hysterectomy.

## Introduction

Postpartum hemorrhage (PPH) remains the leading cause of maternal mortality worldwide, affecting millions of women annually and accounting for over 20% of all reported maternal deaths [1]. The incidence is significantly higher following cesarean delivery (26.5%) compared to vaginal delivery (12.3%) [2]. Uterine atony has been identified as the predominant underlying cause in both modes of delivery.

When medical management with uterotonics fails, conservative surgical interventions are employed to control bleeding while preserving fertility. Among these, uterine compression sutures, particularly the B-Lynch technique, have gained widespread acceptance as effective, uterus-sparing alternatives to hysterectomy [3]. Success rates in avoiding hysterectomy have been reported to exceed 85% [4]. Various modifications, including transverse or square sutures, have also been introduced to enhance compression and control refractory bleeding [3].

Although life‑saving, such procedures can become life‑threatening when combined with devascularization, as reduced uterine blood flow may trigger a cascade from PPH to ischemia, infection, pyometra, and ultimately necrosis, highlighting the critical need for early detection to avert catastrophic maternal outcomes. This case highlights a rare but severe delayed complication following conservative surgical management of PPH, characterized by infected intrauterine collection, uterine necrosis, pelvic hematomas, and chronic anterior abdominal wall sinus formation following B-Lynch compression suturing and bilateral internal iliac artery ligation.

## Case presentation

A 22-year-old primipara underwent induction of labor at term with two doses of intracervical dinoprostone gel, followed by vaginal delivery with a right mediolateral episiotomy 2 and a half months ago. She developed primary PPH immediately after delivery, which could not be managed by uterotonics and eventually required emergency laparotomy with B-Lynch uterine compression sutures along with transfusion of five units of packed red blood cells, followed by intensive care monitoring.

Within 48 hours of surgery, she developed persistent fever and abdominal pain, although her hemodynamic parameters remained stable. On post-natal day (PND) 13, contrast-enhanced magnetic resonance imaging (CEMRI) revealed a postpartum enlarged uterus with large hematoma in both adnexa. Both ovaries were not separately identified. She subsequently underwent re-exploration with hematoma drainage and bilateral internal iliac artery ligation on PND 14.

Despite these interventions, the patient continued to have persistent fever, now with purulent discharge from the abdominal stitch line, and foul-smelling thick vaginal discharge for nearly two months, despite daily wound care and multiple courses of broad-spectrum antibiotics and antifungal therapy. Ultrasonography performed on PND 75 (2 months 15 days) demonstrated a bulky uterus with a residual endometrial lesion and a subcutaneous scar collection with a sinus tract extending to the skin.

The patient was subsequently referred to our institution with complaints of abdominal pain, persistent discharge from the surgical wound and per vaginum, and a palpable abdominal mass. On PND 80 (two months 20 days), the patient was febrile with moderate pallor and had stable vital parameters. Abdominal examination revealed a markedly subinvolution uterus corresponding to approximately 20-week gestational size, which was firm to hard in consistency, immobile, and tender. Per vaginal examination could not be performed as the patient declined consent due to severe pain. All routine and culture investigations were sent, which were within normal limits, except for total leukocyte count, which was 16,700/μL (normal range: 4,000-8,000/μL). Culture from the wound discharge and vaginal discharge were sterile.

On PND 83, contrast-enhanced computed tomography of the abdomen and pelvis demonstrated a large endometrial collection of 10.7 x 9.2 x 6.2 cm with irregular thinning and outpouching of the myometrium with surrounding diffuse extensive inflammatory changes, and a thin tract from the uterine surface to the overlying skin through the anterior abdominal muscle in midline. Features were suggestive of pyometra with sinus tract formation in the anterior abdominal wall (Figures 1A-1B).

**Figure 1:**
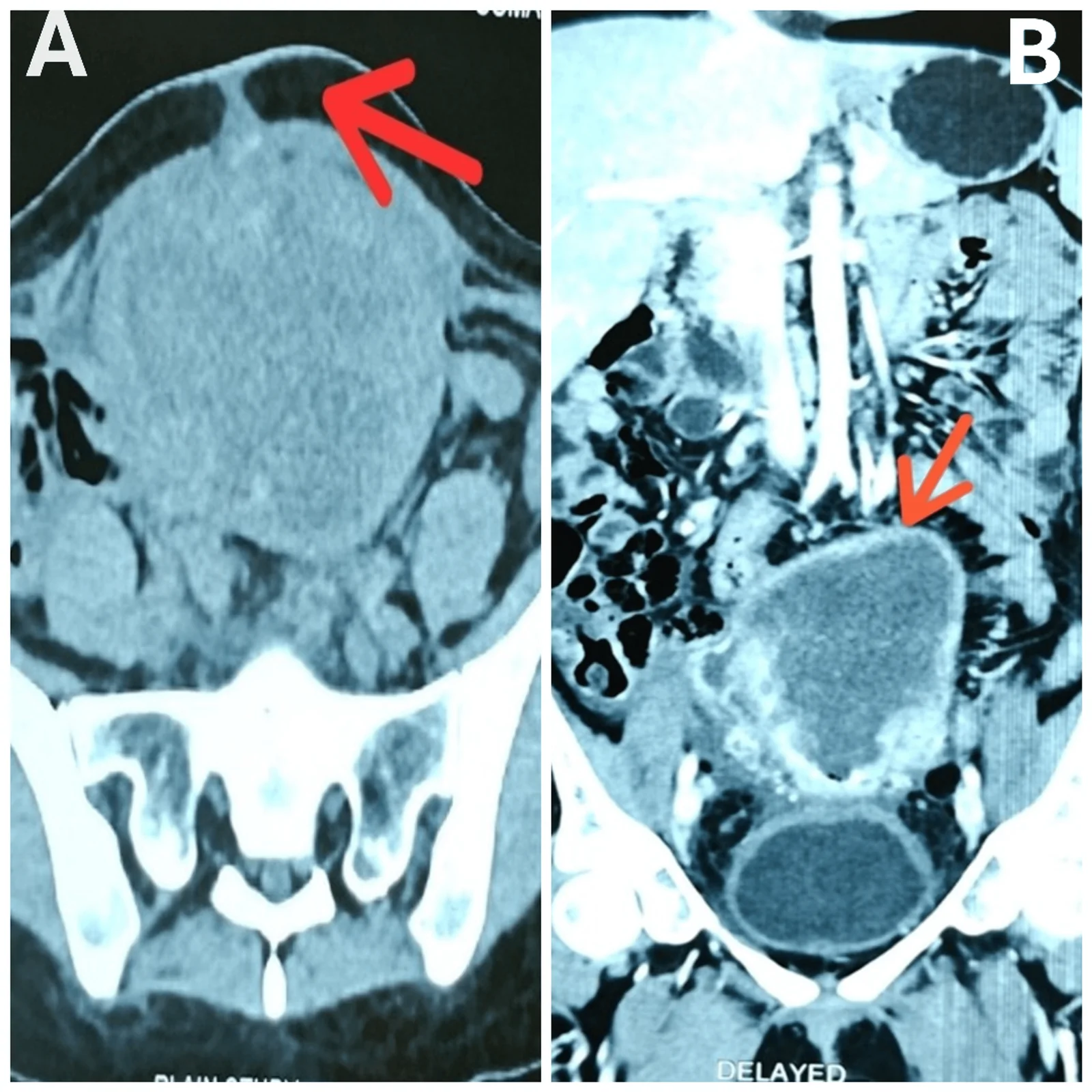
Contrast-enhanced computed tomography (CECT) image of the abdomen and pelvis (1A) Transverse axial view of contrast-enhanced CT showing a sinus tract communicating from the uterus to the anterior abdominal wall (red arrow). (1B) Coronal view of contrast-enhanced CT showing a thinned-out myometrium with a low-density collection in the endometrial cavity (red arrow).

Keeping either infected pelvic hematoma or uterine necrosis with pyometra as differential diagnoses, exploratory laparotomy was planned. The patient underwent exploratory laparotomy on PND 85. Intraoperative findings revealed a small sinus tract ~1 x 1 cm connecting the anterior abdominal wall to the uterus, seen at 2 cm above pubis symphysis. Bowel was densely adhered to the posterior and lateral surface of the uterus. After adhesiolysis, the uterus was found to be completely necrosed to the isthmus and organized pus collection protruding from the fundal region. A few eaten-up absorbable sutures were seen between the walls of the uterus. The lower part of the cervix seems relatively healthy. No adnexal tissue on either side could be appreciated, and it seemed eaten up by necrosis (Figures 2A-2B).

**Figure 2:**
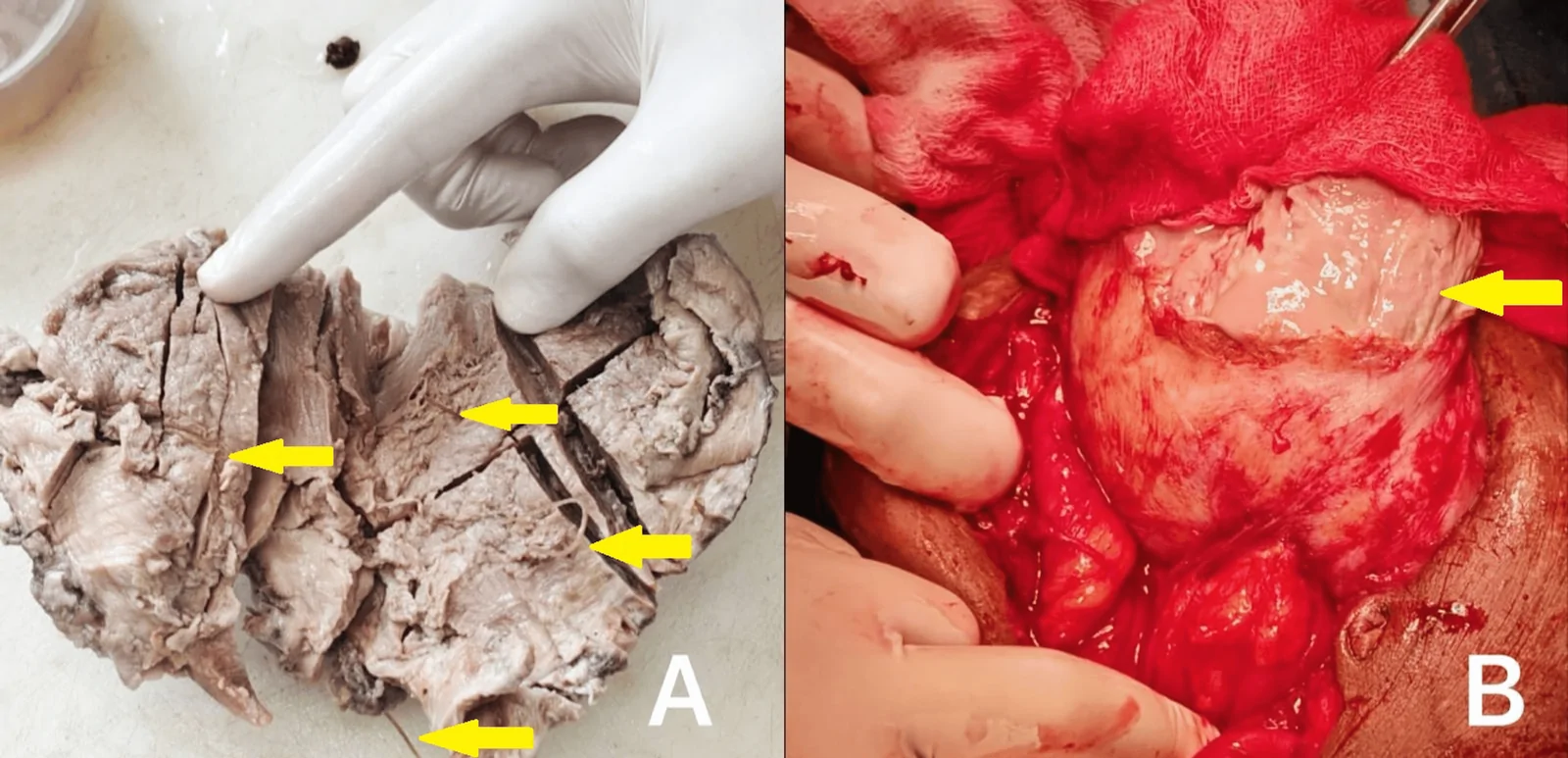
Intraoperative image (2A) Cut-section of the specimen showing B-Lynch sutures (arrow). (2B) Intra-operative image showing the necrosed uterus with organized pus popping out through the myometrium (yellow arrow).

Total hysterectomy was performed. The postoperative period was uneventful except for the surgical site infection, especially poor wound healing at the sinus tract site. The patient was discharged on the 15th postoperative day. On follow-up visit at eight weeks, she was asymptomatic.

## Discussion

PPH is a life-threatening condition, and its management should be fast and possibly conservative. Uterine compression, particularly the B-Lynch technique, has gained widespread acceptance as an effective conservative management option [3]. However, despite their fertility-preserving intent, compression sutures are not without complications. Excessive suture tension or improper placement can compromise uterine perfusion, potentially leading to ischemia and myometrial necrosis [4,5]. Ordinarily, the rapid involution of the uterus helps relieve such tension, and the extensive uterine vascular network further minimizes the likelihood of necrosis. Nevertheless, when additional hemostatic techniques are employed concurrently, they may collectively disrupt the uterine anastomotic network, thereby increasing the risk of necrosis [6]. This mechanism was evident in our patient. While reported incidence rates remain inconsistent across studies, estimates suggest it may occur in approximately 5-7% of cases [7].

Uterine necrosis following B-Lynch sutures typically presents with fever, abdominal pain, foul-smelling discharge, and uterine subinvolution [8]. In the present case too, the patient presented with persistent fever, foul-smelling discharge, abdominal pain, and sinus tract formation, representing a delayed and progressive form of presentation within the same pathological spectrum. Diagnosis is usually suspected on USG and should be confirmed by CECT or MRI. The diagnostic hallmarks of uterine necrosis include the presence of intra-myometrial gas, absence of myometrial enhancement following contrast administration, peripheral contrast uptake, and elongation of the uterine contour [9]. In the present case, the diagnosis was initially suspected on clinical grounds and subsequently confirmed by CECT, which demonstrated pyometra with a sinus tract. Definitive confirmation was obtained intraoperatively and through histopathological examination, establishing the diagnosis of uterine necrosis.

Management in reported cases is predominantly surgical, with most patients requiring hysterectomy due to established necrosis and sepsis [6,8,10]. In this case, hysterectomy was required due to a necrosed uterus with pyometra. Uterine salvage is rarely possible and only in early, pre-necrotic stages [10].

## Conclusions

Uterine necrosis, although a rare and potentially devastating complication, may occur following the use of uterine compression sutures and/or devascularization procedures when uterine perfusion is critically compromised. Excessive or prolonged vascular occlusion, combined with excessive tissue compression, may impair both arterial inflow and venous drainage, resulting in progressive tissue ischemia and necrosis. This case highlights the importance of meticulous surgical technique, appropriate tension and placement of compression sutures, and vigilant postoperative monitoring for early signs of uterine ischemia to prevent such severe outcomes.
